# A Deep Learning Framework for Automatic Meal Detection and Estimation in Artificial Pancreas Systems

**DOI:** 10.3390/s22020466

**Published:** 2022-01-08

**Authors:** John Daniels, Pau Herrero, Pantelis Georgiou

**Affiliations:** Centre for Bio-Inspired Technology, Department of Electrical and Electronic Engineering, Imperial College London, London SW7 2AZ, UK; pherrero@imperial.ac.uk (P.H.); pantelis@imperial.ac.uk (P.G.)

**Keywords:** type 1 diabetes, meal detection, carbohydrate estimation, artificial pancreas, multitask learning, machine learning, deep learning, neural network, quantile regression

## Abstract

Current artificial pancreas (AP) systems are hybrid closed-loop systems that require manual meal announcements to manage postprandial glucose control effectively. This poses a cognitive burden and challenge to users with T1D since this relies on frequent user engagement to maintain tight glucose control. In order to move towards fully automated closed-loop glucose control, we propose an algorithm based on a deep learning framework that performs multitask quantile regression, for both meal detection and carbohydrate estimation. Our proposed method is evaluated in silico on 10 adult subjects from the UVa/Padova simulator with a Bio-inspired Artificial Pancreas (BiAP) control algorithm over a 2 month period. Three different configurations of the AP are evaluated -BiAP without meal announcement (BiAP-NMA), BiAP with meal announcement (BiAP-MA), and BiAP with meal detection (BiAP-MD). We present results showing an improvement of BiAP-MD over BiAP-NMA, demonstrating 144.5 ± 6.8 mg/dL mean blood glucose level (−4.4 mg/dL, p< 0.01) and 77.8 ± 6.3% mean time between 70 and 180 mg/dL (+3.9%, p< 0.001). This improvement in control is realised without a significant increase in mean in hypoglycaemia (+0.1%, p= 0.4). In terms of detection of meals and snacks, the proposed method on average achieves 93% precision and 76% recall with a detection delay time of 38 ± 15 min (92% precision, 92% recall, and 37 min detection time for meals only). Furthermore, BiAP-MD handles hypoglycaemia better than BiAP-MA based on CVGA assessment with fewer control errors (10% vs. 20%). This study suggests that multitask quantile regression can improve the capability of AP systems for postprandial glucose control without increasing hypoglycaemia.

## 1. Introduction

The self-management of diabetes is a burdensome, delicate, and yet critical undertaking that individuals with diabetes have to engage in daily in order to avert adverse glycaemic events. Current diabetes management systems such as the artificial pancreas (AP) and decision support systems have been developed in recent years in order to improve the management of diabetes. The artificial pancreas is a system that comprises a continuous glucose monitor (CGM), insulin pump, and an algorithm working in tandem to maintain blood glucose levels in an acceptable range (70–180 mg/dL). The envisaged endpoint in the development of the insulin-based artificial pancreas is a fully automated system that does is not depend on user input throughout the day [1]. One major challenge in the realising this objective with the artificial pancreas centres on postprandial glucose control. Studies have shown that when small meals (e.g., 30 g) are missed AP systems are capable of handling the resulting postprandial increase in glucose [2,3]. However, control is noticeably poorer with larger sized meals [2,3]. As a result, the current artificial pancreas systems is classified as a hybrid closed-loop system since it requires meals to be announced prior to mealtime in order to ensure good control.

The need for user attention to initiate meal announcements in hybrid closed-loop systems can lead to sub-optimal outcomes. In the ideal scenario, the user accurately estimates the meal size by carbohydrate counting and provides that information to the AP system prior to eating. This has to be done due to the delays associated with subcutaneous insulin delivery. However, multiple studies have shown that individuals with diabetes tend to have significant rates of late or missed meal insulin boluses [4]. This can be attributed to factors such as diabetes distress, stress and forgetfulness among others in a daily routine [5,6,7]. These studies generally showed a strong link between late and missed meal boluses and HbA1c levels. This increase in HbA1c levels can lead to worsening quality of life over time [8,9,10,11,12]. Furthermore, individuals with diabetes are reported to misestimate meal sizes between 20% and 59% [13,14].

This appropriately motivates the need for systems such as the AP to be able to detect unannounced meals and estimate the carbohydrate size in order to control postprandial hyperglycaemia.

The rest of the paper is organised as follows: Section 2 presents an overview of the broad categories in the literature for tackling meal detection and some proposed methods under each category, thus providing the context for our work. In Section 3, we provide a detailed description of the neural network architecture and proposed framework for detecting and estimating unannounced meals. This builds on preliminary work towards this task [15]. We also detail the simulation scenario generated by the UVa/Padova simulator for evaluating the proposed framework. Section 4 presents the results on meal detection and estimation along with the results comparing our proposed fully closed-loop controller against baseline configurations of the closed-loop controller. Section 5 comprises a discussion of the earlier presented results in comparison to the related works and elaborates on the implications of uncertainty quantification towards safely closing the loop in glucose control. This sets the ground for future work in the area.

## 2. Related Work

A number of methods have been proposed in the literature towards tackling the detection and estimation of unannounced meals. These methods broadly fall into the following categories: (i) threshold-based detection using the rate of change (ROC) of glucose levels and (ii) outlier detection using model predictions.

Various threshold-based approaches are proposed that analyse a number of rules regarding the rate of change for meal detection. Dassau et al. [16] propose an ensemble approach that comprises three or four approaches -the backward difference (BD), the second derivative of glucose, a Kalman filter (KF) estimation, and a combination of BD and KF -with meal detection dependent on attaining a majority vote from the approaches. Ramkisson et al. [17] employ an unscented Kalman filter that extends Bergmans minimal model to account for a disturbance parameter and tracks a cross-covariance of the forward difference of this parameter and glucose level, and the rate of change to confirm meal detection. To include meal size estimation, Samadi et al. [18,19] initially analyse the first and second derivatives of a filtered CGM signal in order to detect a meal, and then estimates the meal size from glucose levels and insulin-on-board (IOB) using a fuzzy logic system.

A potential downside of relying on the rate of change and pre-defined thresholds for meal detection is that when there is significant variability in glucose levels this would lead to a reduction in the signal-to-noise ratio, and consequently, a relatively high rate of false positives which can be potentially harmful in this context [18,19]. As a result, some approaches typically require filtering which may then increase the detection delay and reduce sensitivity. Even then, the thresholds are selected based on a training dataset with specific underlying conditions (e.g., sensor noise, sensor drift, insulin sensitivity), so when these conditions shift significantly over the deployment period this may lead to degraded performance while going undetected.

The alternative approach is model-based approaches that then detect meals based on outliers in the glucose trajectory for which various approaches have been proposed as well [20,21,22]. Cameron et al. [20] develop a probabilistic approach that compares the expected signal and observed signal to detect a meal, and with assumptions on the meal shapes, estimate the glucose rate of appearance. A variable state dimension (VSD) approach is introduced where an extended Kalman filter (EKF) is used to predict the glucose trajectory with a 95% prediction interval [21]. A meal is detected, once the upper bound is exceeded, and a least-squares approach estimates the meal size. Mahmoudi et al. propose a similar approach that instead uses an unscented Kalman filter (UKF) to predict the glucose trajectory [22]. However this work employs two CGM sensors as opposed to one CGM sensor, and a meal is detected once both glucose trajectory from both sensors exceed the 95% prediction interval. Zheng et al. [23] employ the minimal model and run multiple simulations when the difference between the model trajectory and CGM trajectory is larger than a set value, to explain the likely meal size to reduce this divergence. Finally, Garcia-Torado et al. [24] rely on a classification model (logistic regression) that continuously estimates the probability of an unannounced meal and provides a scaled insulin bolus based on the total daily insulin instead of estimating the carbohydrate content.

This work devises a data-driven approach that leverages a deep multitask learning framework in order to detect and estimate meals. To the best of our knowledge this is the first meal detection and estimation framework based on multitask quantile regression, where the assumption on distribution of errors is relaxed. As a result we make the following contributions:

- We develop a novel meal detection algorithm based on multitask neural networks and quantile regression in order to automatically announce meals and estimate meal size.
- We evaluate in silico the performance of the meal detection and estimation algorithm in moving towards fully closed-loop insulin delivery.

## 3. Materials and Methods

In this section, we first present a meal detection and estimation algorithm that is based on a sequence to sequence model that is extended to a multitask setting in order to perform multiple quantile regression. We first describe the recurrent neural network that makes up this framework. This method is evaluated using a in silico dataset of 10 adult subjects generated through the UVa-Padova simulator. We then illustrate the use case in the AP setting and present results on in silico validation.

### 3.1. Multitask Deep Neural Network

As mentioned earlier, the deep neural network is based on a multitask sequence-to-sequence model. The sequence-to-sequence (seq2seq) model is primarily a model that is used to map one set of input sequences to an associated set of output sequences and has been used in glucose prediction tasks [25,26,27]. In this task, the objective is to estimate the last 20 min of the individual’s glucose trajectory using historical CGM measures, meals, and insulin.

**Figure 1:**
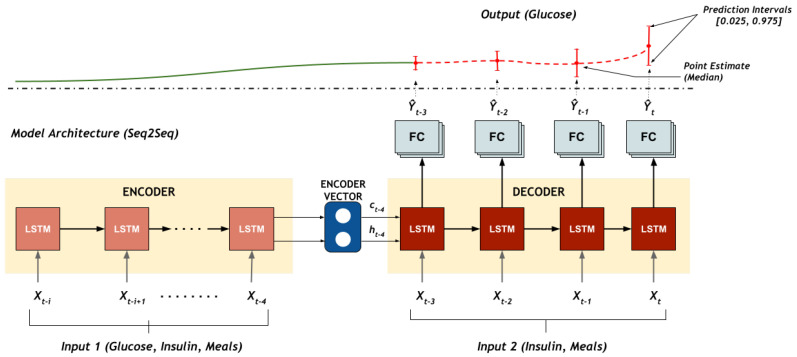
The multitask deep neural network architecture for predicting the 20 min glucose trajectory at multiple quantiles. The multitask seq2seq model predicts glucose trajectory with a 95% prediction interval (PI) with a lower bound (2.5%) and upper bound (97.5%).

To fully utilise the information available, a recurrent neural network encoder-decoder architecture shown in Figure 1 is employed in this framework to estimate the glucose trajectory. In this model, the encoder and decoder are based on long short-term memory (LSTM) networks due to their ability to better model sequential data without the issue of vanishing gradients [28]. The encoder LSTM input sequence—$x_{enc}$ = $\left(x_{t-i},...,x_{t-4}\right)$—comprises the glucose concentration levels from the CGM, insulin delivered and meals and returns the encoder state representations {c, h}. The decoder generates the output sequence—$y_{dec}$ = $\left(y_{t-3},...,y_{t}\right)$—and estimated quantiles of the glucose trajectory are generated at the output. The decoder input, $x_{dec}$ = $\left(x_{t-3},...,x_{t}\right)$, comprises the insulin delivered and announced/estimated meals and is initialised with the final encoder state.

The output sequence generated from the decoder is then fed to the three final layers of the model that consists of three separate tasks. Each task represents a quantile distribution, τ, and consequently the model performs a quantile regression for the associated quantile.

#### 3.1.1. Quantile Regression

Quantile regression can be defined as method of estimating a conditional quantile, τ, where τ∈⌈0,1⌉ for a target variable, Y conditioned on the input, X [29]. Subsequently, the resulting prediction interval serves as an approach for estimating the aleatoric uncertainty in the training data. One approach that we utilise in training a deep neural network to perform quantile regression is the pinball loss/tilted loss as shown in Equation (1).

(1) $$L_{\tau }\left(y\left(t\right),\hat{y}\left(t\right)\right)=\left\{\begin{array}{cc} \tau (y\left(t\right)-\hat{y}\left(t\right)), & y\left(t\right)-\hat{y}\left(t\right)>0,\\ \left(1-\tau \right)(y\left(t\right)-\hat{y}\left(t\right) & \mathrm{otherwise}, \end{array}\right.$$

where *y* refers to the reference glucose value, $\hat{y}$ refers to the predicted value, and τ refers to the quantile that the regression is estimating.

Aleatoric uncertainty captures the uncertainty in the available data for training a model. This uncertainty may arise due to errors or variability in the data. In this case the errors may arise from the misestimation of meals or noise in measurements of glucose concentration levels from the CGM. As a result, once a persistent deviation from the prediction interval is noted this significant disturbance can be attributed to an unannounced meal. This forms the basis of the proposed approach to the meal detection and estimation algorithm in this work.

Typically the deep learning model is trained using a mean absolute loss or mean squared loss [30]. The mean absolute loss minimises the sum of absolute differences between the predicted value and the target value to perform a regression. Notably, we note from Equation (1) that at the median quantile (τ = 0.5), the quantile loss is equivalent to the mean absolute loss. In this work, we extend the utility of the deep learning model to output the glucose trajectory of multiple quantiles.

In our multitask architecture, each output corresponds to the following quantiles: A lower bound quantile ($\tau_{LB}$), a median quantile ($\tau_{M}$ = 0.5), and an upper bound quantile ($\tau_{UB}$). The total of the quantile losses, $L_{total}$, are uniformly weighted and jointly minimised as seen in Equation (2).

(2) $$L_{total}=\frac{1}{3}\left\lceil L_{LB}+L_{M}+L_{UB}\right\rceil$$

**Figure 2:**
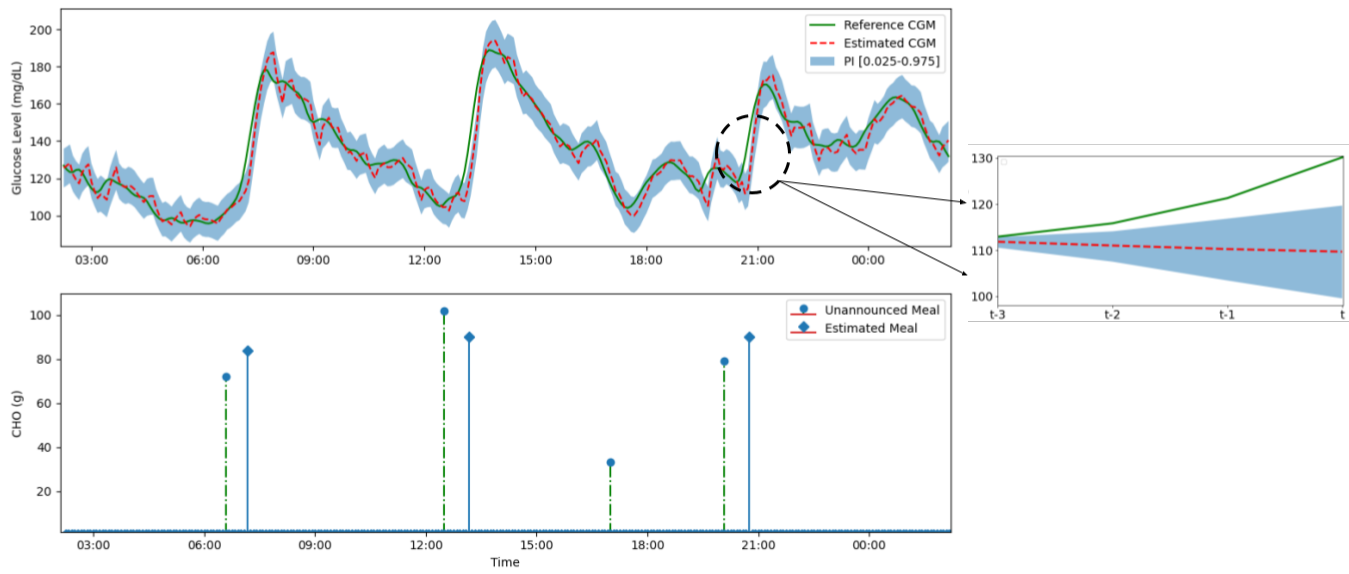
(**Top**) A figure showing the multitask seq2seqmodel predicting the 20 min glucose trajectory for a 95% prediction interval (lightly shaded blue area) over a 24-h period. The estimated glucose level (red dashed line) closely follows the reference glucose level from the CGM (green line) except during unannounced meals. In the instance of an unannounced meal—such as dinner just before 9 p.m.—a significant and persistent deviation in glucose trajectory from estimated trajectory occurs (shown on the right) which leads to a meal detection and estimation. (**Bottom**) The unannounced meal and reconstructed estimated meal. Certain meals such as the small snack between 3 p.m. and 6 p.m. are not detected as reliably since they only result in a non-significant deviation in the glucose trajectory.

The multitask architecture is beneficial in terms of computation and memory since the majority of weights are shared as opposed to having independent models for each quantile. Furthermore, Tagasovska et al. show that using a multitask architecture also enables consistent uncertainty quantification when modelling inherent noise in data [31]. Figure 2 below shows that consistent prediction interval of the from our multitask model for an adult subject. Further results showing the accuracy in obtaining the pre-specified prediction intervals is detailed in Appendix A.

#### 3.1.2. Network Training

Prior to training the input features are first normalised. The neural network for each individual is trained in a two step strategy. The individual dataset is split into 80% training data and 20% validation data. In order to attain better performance we first train a generalised model using aggregated data from each individual. The individualised model for each participant is then obtained by fine-tuning the generalised model on the individual data.

The LSTM layers for both the encoder layer and decoder layer consist of 64 cells. The selected optimiser is the Adam optimiser and at the pre-training stage the learning rate is $1\times 10^{-3}$, which is then reduced to $1\times 10^{-4}$ at the fine-tuning stage. The batch size for both training stages is 128. We set the number of epochs to 100 and implement early stopping with a patience of 20 epochs to terminate training when validation loss is no longer improving ($\Delta L_{min}=1\times 10^{-4}$). The models are developed with Python 3.6 and Keras v2.2.2 and trained using a NVIDIA GTX 1050.

### 3.2. Meal Detection and Estimation

#### 3.2.1. Meal Detection

The first stage of the model involves the detection of a meal. First, the last 20 min of the glucose profile is estimated using the sequence to sequence model. As explained in the previous section, the model outputs multiple quantiles for a 95% prediction interval coverage that arises from the errors arising from the input variables. Consequently, in a scenario where the glucose trajectory persists outside the prediction interval, this implies a significant deviation beyond noise in the input variables that can be inferred as a missing input. In this setting, this missing input is attributed to an unannounced ingested meal and therefore a meal detection flag can be activated.

The use case of this framework necessitates the priority of safety. As a result, in the detection of the algorithm, we set the condition that this deviation needs to persist for at least 3 samples (15 min) to satisfy activating the meal detection flag. In addition, the flag is only activated if the rate of increase of glucose trajectory is at least 1 mg/dL/min.

#### 3.2.2. Carbohydrate Estimation

Once the meal detection flag is activated we begin the process of estimating the meal size. This step is achieved with a simple iterative search approach implemented at the input in order to determine the best possible meal size to estimate the present CGM value. We condition this iterative search on the mean absolute error (MAE) between the median quantile of the present estimated glucose level and the reference glucose level from the CGM. The search is completed once the mean absolute error is less than a threshold, ϵ. We initially increase the meal input at 10 g and review the mean absolute error (MAE) until the result is lower than the threshold. We then reduce the meal increment to 1 g and rerun the last iteration to obtain a more precise meal size estimate. To mitigate serious effects of overestimation the maximum meal size for an instant is limited to $m_{MAX}$= 90 g. Additional successive meal estimates can be made to supplement the initial estimate

(3) $$\begin{array}{cc} \epsilon & =MAE_{val}\\ \; & =\frac{1}{N}\sum_{k=1}^{N}\left|y\left(k\right)-{\hat{y}}_{M}\left(k\right)\right|, \end{array}$$

where ${\hat{y}}_{M}\left(k\right)$ denotes the predicted glucose level for a given sample at the media quantile, *k*. y(k) denotes the reference glucose measurement, *N* refers to the number of samples in the validation set.

For robustness and verification, a check is included where if the meal size increment does not lead to the expected physiological increase in trajectory (an increase in MAE), the meal is discarded as this violates the expected physiological dynamics. The algorithm pseudocode is detailed in Algorithm 1 below. On a 24 h glucose profile, a graphical representation of Algorithm 1 of the detection and estimation of meals is shown.

### 3.3. Fully Closed-Loop Control for Insulin Delivery

A number of closed-loop controllers have been proposed in the literature in order to facilitate tight glycaemic control [1,32]. Examples of the different types include proportional-integrative-derivative (PID), model predictive control (MPC), reinforcement learning-based (RL) controllers, and bio-inspired controllers. The bio-inspired artificial pancreas (BiAP) is a hybrid glucose controller based on that has been validated in previous work [3] and is employed in this work to evaluate the meal detection and carbohydrate estimation algorithm performance. It should be noted, however, that this proposed algorithm is agnostic to the choice of controller.

**Figure 3:**
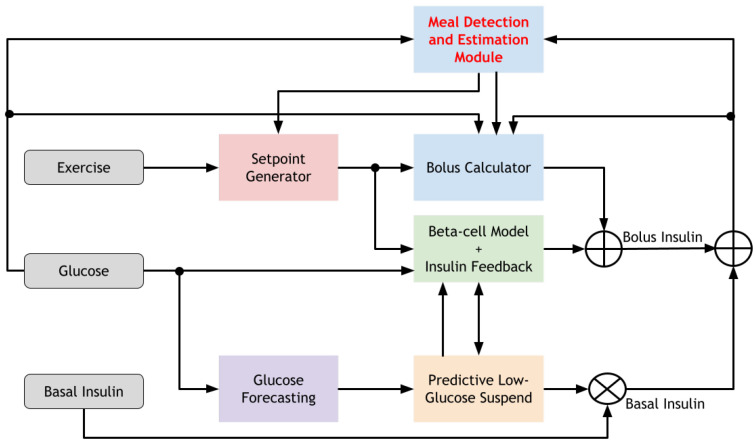
The system architecture of the bio-inspired artificial pancreas with the meal detection and estimation algorithm incorporated.

As shown in Figure 3 the meal detection and estimation module provides an estimate of the carbohydrate size to the bolus calculator in the BiAP controller to determine the meal insulin bolus. However, because of the increase in glucose levels due to the meal ingestion, the controller begins to deliver insulin to cover the meal. This insulin is delivered as the deviation remains below the upper bound and the meal flag is not raised. In addition the meal size estimate may be overestimated. The prudent measure to cover these scenarios is to remain conservative and minimise the possibility of precipitating a late postprandial hypoglycaemic event. Consequently a weight (W = 0.5) is applied to the calculated meal bolus, and no correction bolus to compensate for hyperglycemia is applied together with the meal bolus. Furthermore, the basal insulin delivery is suspended for 1 h after the meal bolus is delivered.
**Algorithm 1** Meal Detection and Estimation Algorithm**Require:**X={G,I,M}, ϵ**Ensure:** Output meal size, $m_{E}$
 Initialise model parameters θ from memory N←2 **for**
t∈28,...,T
**do**     $[y_{u},y_{m},y_{l}]\leftarrow$
f(X|θ)     Compare $y_{u}$ and $y_{CGM}$     Let k be number of samples where $y_{u}$< $y_{CGM}$     **if** k > N AND
ΔBG/Δt ≥ 1 **then**         ▹ Activate meal detection
          $m_{E}\leftarrow 0$
          $error\leftarrow \mathrm{Abs}(y_{m}\left(t\right)-y_{CGM}\left(t\right))$
          **while** error >ϵ
AND
$m_{E}<m_{MAX}$
**do**      ▹ Perform meal estimation
              **if** fine_search **then**
                  $m_{E}\leftarrow m_{E}+1$
              **else**                  $m_{E}\leftarrow m_{E}+10$               **end if**              $M\leftarrow m_{E}$              $[y_{u},y_{m},y_{l}]\leftarrow$
f(X|θ)              $error\leftarrow \mathrm{Abs}(y_{m}\left(t\right)-y_{CGM}\left(t\right))$              **if** error ≤ϵ **then**
                  $m_{E}\leftarrow m_{E}-10$                  Activate fine_search              **end if**              **if** verify appropriate glucose dynamics **then**                  $m_{E}\leftarrow 0$     ▹ Discard meal estimate                  **break**              **end if**          **end while**     **else**           $m_{E}\leftarrow 0$     **end if****end for**

### 3.4. Performance Metrics

We employ multiple metrics to comprehensively evaluate the meal detection and estimation framework. To assess the detection of meals we use the following metrics: precision, recall, F-score, and detection time delay.

(4) $$\mathrm{Precision}=\frac{\mathrm{TP}}{\mathrm{TP}\;+\;\mathrm{FP}}$$

(5) $$\mathrm{Recall}=\frac{\mathrm{TP}}{\mathrm{TP}\;+\;\mathrm{FN}}$$

A true positive (TP) is identified when the detection flag is raised and the delay in identifying the meal is less than 120 min. Otherwise, this event is identified as a false negative (FN). A false positive (FP) is identified when a meal flag is raised in the absence of a meal. In the eventual use case of a meal detection algorithm, the detection of an unannounced meal would prompt a bolus to be delivered either indirectly by notifying the user with an alert, or directly in a sensor augmented pump. Subsequently, it is important that the metrics not only assess the effectiveness of the algorithm in detecting unannounced meals, but also we assess the quality of the meal detection. The recall (sensitivity) assesses the ability of the algorithm is detect a meal, whereas the precision assesses the quality of meal detection. For the meal detection time, we evaluate based on the median delay between the detected meals and the actual meals. For assessing the effectiveness of *CHO* estimation, we analyse the meal size errors and percentage error.

The performance of glycaemic control is evaluated using a comprehensive set of metrics that are typically used in the literature [33]. We primarily report the following glycaemic metrics: percentage time spent in euglycaemia (70 mg/dL < BG < 180 mg/dL), percentage time spent in hyperglycaemia (BG ≥ 180 mg/dL), percentage time spent in hypoglycaemia (BG ≤ 70 mg/dL), mean glucose concentration level. We evaluate the level of control with a number of indices, particularly, the high blood glucose index (HBGI), low blood glucose index (LBGI), and risk index (RI).

Finally, we provide a visual comparison of the quality of closed-loop glycaemic control of the different configurations with a control-variability grid analysis (CVGA) [34]. This visualisation is complemented with a numeric assessment of the quality of control.

#### Statistical Analysis

We evaluate the differences in the controller performance with different configurations: meal announcement, meal detection, and unannounced. For determining the statistical significance, we first perform a preliminary test for normality using a Shapiro–Wilk test. We use a paired *t*-test if normality is accepted, and a Wilcoxon signed-rank test when normality is rejected. Significance level is set at *p*-value < 0.05. For multiple pairwise comparisons, we adjust the significance level to *p*-value < 0.025 using Bonferroni correction. The data from the results are presented as Mean ± SD.

### 3.5. In-Silico Dataset

The University of Virginia/Padova (UVa/Padova) T1D Simulator is used to generate a challenging scenario for training, validating and testing the models. For the meal protocol scenario we choose four meals with the following average carbohydrate size at the associated average meal times: 70 g (7 a.m.), 100 g (1 p.m.), 30 g (5 p.m.), and 80 g (8 p.m.). A meal-time variability ($\sigma_{T}$ = 60 min) and meal size variability (CV = 10%) is introduced in order to generate realistic scenario of inter-day variability in meals. In addition, to account for additional variability in meal composition, the simulator meal library was supplemented with a further 16 new meals as described by the authors in [35].

To generate more realistic scenarios, we modify the original version of the simulator to introduce additional intra-day variability on insulin absorption and insulin sensitivity. Variability of insulin absorption is assumed to be ±30% and the insulin sensitivity varies in a sinusoidal manner with a selected daily period.

As mentioned earlier, studies have shown that individuals with diabetes are not always prompt with meal announcements and also the carbohydrate counting is consistently misestimated. In order to model this behaviour, a trigger for skipping and delaying meal announcement was randomly generated based on an average 2.5 meal announcements skipped, and 2 meals delayed per week [10]. Carbohydrate counting uncertainty is incorporated in the simulator [−30%, +10%] with a uniform distribution. The values are selected due to the bias towards underestimation rather than overestimation of carbohydrate size. These parameter choices are then used consistently across all tests and comparisons.

As noted earlier, a 2-month scenario is generated for training and validating the models in an offline setting. Furthermore, the duration of the simulation scenario in testing the proposed algorithm is also 2 months however, this is executed in an online setting.

## 4. Results

### 4.1. Meal Detection and Estimation Performance

In this section, we report the results on the performance of the framework in detecting and estimating the unannounced meals in the simulation scenario. The performance is reported on each mealtime and for the snacks.

Table 1 shows the meal detection performance of the meals and snacks at different mealtimes. The average breakfast meal size is of moderate size (70 ± 7 g). The method obtains a precision 86 ± 7% and a recall 90 ± 5%. In terms of detection time these meals are flagged at 38 ± 13 min. The lunch meals are the largest size considered in this study at 100 ± 10 g. At lunchtime, the proposed method detects lunch with 98 ± 2% precision and 97 ± 3% recall. This detection is completed in 37 ± 15 min. Finally, for dinner, the average size during this mealtime is 80 ± 7 g which can also be considered moderate-sized. For this proposed method, we detect dinner meals at 94 ± 5% precision and 89 ± 4% recall. The snacks ingested after lunch are of a relatively smaller size at 30 ± 3 g. Although the precision is high at 97 ± 5%, the recall is relatively low at 24 ± 15%. In addition, the detection time is 41 ± 23 min. The observation is made that the recall and detection time performance is dependent on the size of the meal, where small meals (snacks) show the worst performance and large meals (lunch) show the best performance, with moderately sized meals (breakfast and dinner) showing intermediate performance. On the other hand, we notice that the performance in terms of precision is consistently high (86–98%) across different meal sizes.

**Figure 4:**
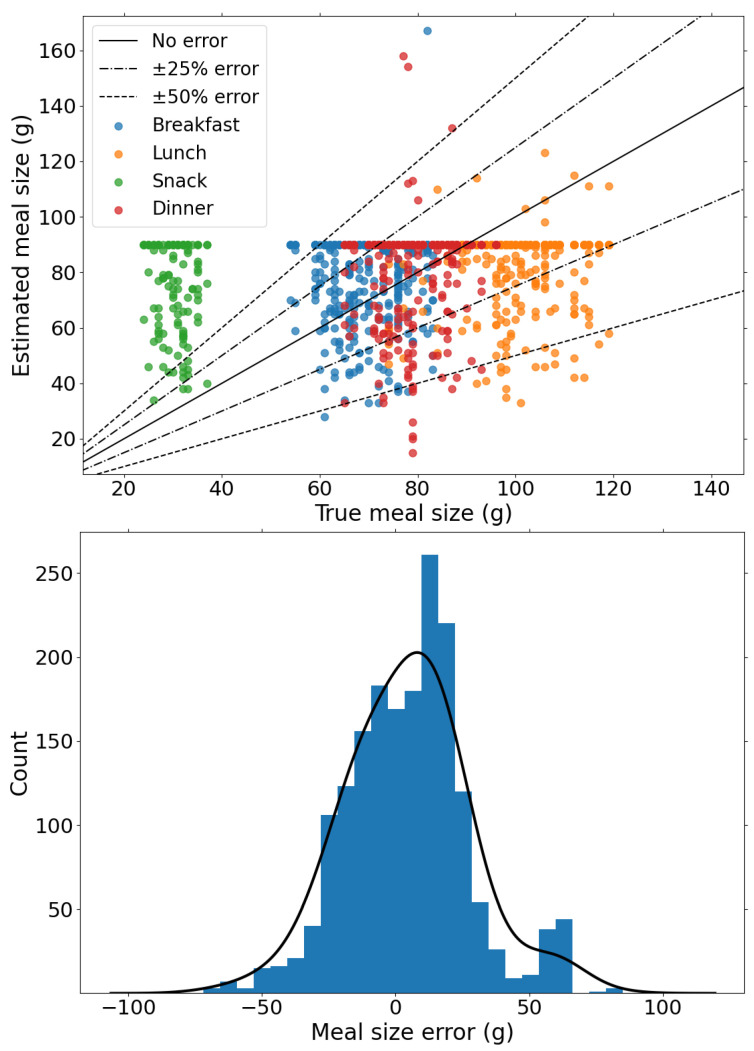
(**Top**) Estimated meal size versus true meal size for detected meals to determine the accuracy of meal size error. (**Bottom**) The distribution of estimated meal sizes and true meal sizes. The probability densities of meal and snack size errors is also shown.

Following the evaluation of the meal detection performance, we analyse the performance of the proposed method on meal estimation. Figure 4 shows the comparison of the estimated meal size and the actual meal sizes and shows the distribution of estimation errors in meals. First, we note that carbohydrate estimation is performed in relation to the median glucose level trajectory which have varying errors and thus can lead to under- and overestimation of meals. In total, 80% of the estimated CHO have a estimation error within 25 g, whereas only 6% of detected CHO is larger than 50 g, of which snacks represent the majority. The distribution of the estimation errors, shown in Figure 4, shows that the proposed method is slightly biased towards overestimation with an average error 18 ± 15 g. This justifies applying the weight to the mealtime insulin bolus for the insulin delivery strategy to mitigate postprandial hyperglycaemia without significantly increasing hypoglycaemia risk.

### 4.2. Closed-Loop Glucose Control

In this section, we report results of the performance of the BiAP controller with different configurations. The different configurations used in this study are described below:

BiAP-NMA: In this configuration, meals are not announced prior to the selected mealtimes for bolus priming. The controller is therefore only able to respond to the postprandial glucose excursion through feedback from the CGM signal. Since there is no external input from the user for meal announcement this is a fully closed-loop configuration.

BiAP-MD: This is a fully closed-loop configuration that corresponds to the BiAP controller with the meal detection and estimation module incorporated. In this configuration, the insulin bolus is delivered as explained in the closed-loop insulin delivery.

BiAP-MA: This hybrid closed-loop configuration corresponds to the controller with meal announcement included. Meal announcement involves the individual estimating the meal size and input this in the controller in order to deliver a preprandial insulin bolus. The behaviour of the individual is modelled as earlier described to account for carbohydrate misestimation, missed boluses and late boluses.

The performance of the controllers in enabling tight glycaemic control is reported in Table 2. BiAP-MD and BiAP-NMA are the two closed-loop controllers that are described as fully closed-loop. A comparison of the performance between these two controllers reveals that the meal detection and estimation algorithm improves the control of postprandial hyperglycaemia. This is evident from the significant reduction in time spent in hyperglycaemia (ΔTAR = −4.2%, p=0.0009) and lower risk of hyperglycaemia (ΔHBGI = −0.8%, p=0.0005). Overall, BiAP-MD reports a significantly lower mean glucose level (−4.4 mg/dL, p=0.003) than the BiAP-NMA controller and provides relatively tighter glycaemic control (ΔTIR = +3.9%, p=0.0007). Finally, this is accomplished without a statistically significant increase in time spent in hypoglycaemia (ΔTBR = +0.1%, p=0.4) or risk of hypoglycaemia (ΔLBGI = +0.1, p=0.1).

**Figure 5:**
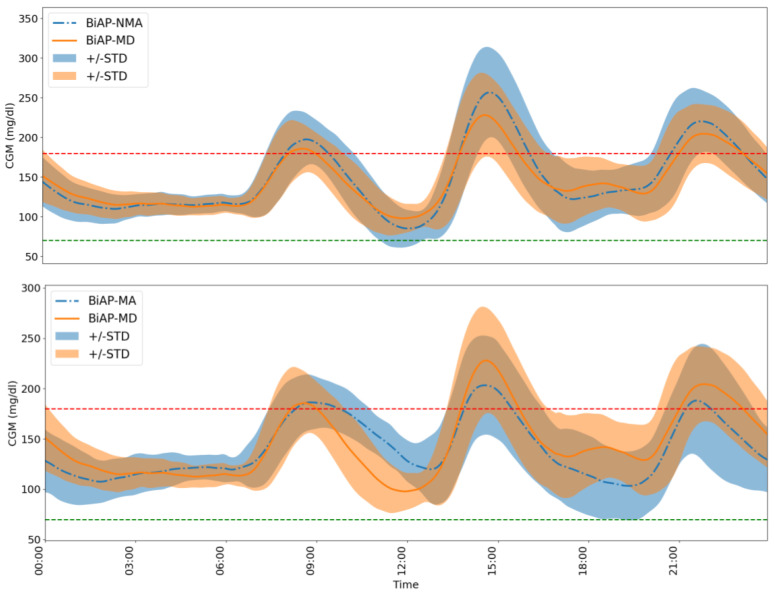
A comparison of the 24 h glucose profile of Adult 3 from the virtual cohort over the 2-month period between the BiAP controller with meal detection and the two baseline configurations. The red and green lines mark the hyperglycaemia and hypoglycaemia threshold, respectively. (**Top**) BiAP-NMA vs. BiAP-MD:A comparison between the BiAP controller performance without and with the meal detection and estimation incorporated. (**Bottom**) BiAP-MA vs. BiAP-MD: A comparison between the BiAP controller performance with user-initiated meal announcement and automatic meal detection and estimation.

A further comparison is made between the BiAP-MA and BiAP-MD controller. The first observation is that BiAP-MA has lower mean blood glucose level (−6.8 mg/dL, p=0.002) than BiAP-MD. In addition, we see significant improvement in tight glycaemic control with BiAP-MA over the proposed BiAP-MD controller: increased time in range (+6.9% mg/dL, p=0.002), reduced time spent in hyperglycaemia (−7%, p=0.002), and reduced associated risk of hyperglycaemia (−1.1, p=0.002). This difference in performance highlights the advantage of the individual pre-bolusing for meals over automatic meal detection and estimation. The accumulation of errors in meal announcements may lead to an increase in time spent in hypoglycaemia (+0.1%, p=0.8) and associated risk of hypoglycaemia (+0.1%, p=0.8), however, these are not observed to be statistically significant. The difference in 24 h glucose profiles of an individual using the BiAP controller with the different configurations over the 2-month period is highlighted in Figure 5.

**Figure 6:**
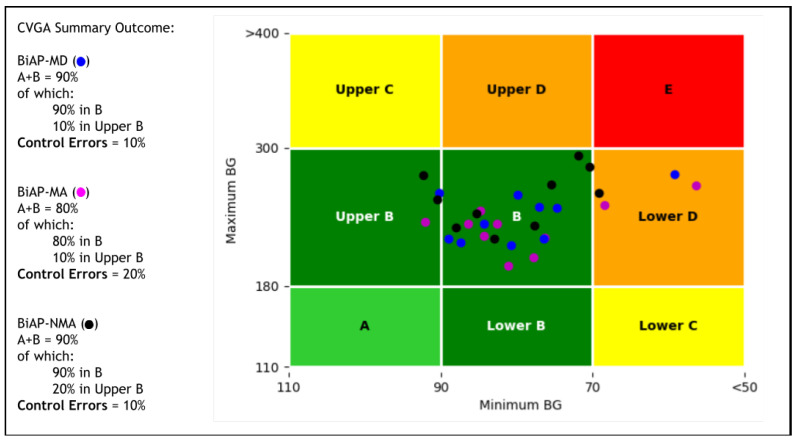
Control variability grid analysis for the BiAP controller with the different configurations reported. Each marker represents a virtual adult in the simulation assessed for the 2 month period.

An analysis of the CVGA plots on the population level as shown in Figure 6 reveals a difference in the quality of glycaemic control for the different configurations. The first comparison we consider is between the fully closed-loop BiAP controllers (BiAP-NMA and BiAP-MD). The general numerical assessment shows that both configurations demonstrate a similar performance with 90% in Zone A+B and 10% in Lower D zone. In detail, however, the observation seen in Table 2 BiAP-MD exhibits tighter glycaemic control than BiAP-NMA is further buttressed in this plot. In total, 10% of BiAP-MD markers were observed in the Upper B zone which is an improvement in comparison to 20% of BiAP-NMA markers, therefore displaying a lesser tendency towards benign control deviations into hyperglycaemia.

On the other hand, for the second comparison we consider quality of glycaemic control between BiAP-MA (hybrid closed-loop configuration) and BiAP-MD (fully closed-loop configuration). BiAP-MA shows marginally worse quality control with 80% of the population in Zone A+B compared to BiAP-MD with 90% in Zone A+B. As seen in Table 2 earlier, BiAP-MA provides tighter glycaemic control that BiAP-MD, however, this can is more likely to lead to more instances of hypoglycaemia particularly when meals are overestimated and delayed. This would explain the higher instance of individuals from the population in Lower D zone and therefore a failure to deal with hypoglycaemia during control.

## 5. Discussion

### 5.1. Comparison with Other Approaches

As discussed in the related works section, a number of methods have been proposed towards detection and estimation of unannounced meals. These generally come under Kalman filters and/or heuristic rules. The reported metrics of the approaches are shown in Table 3 below.

We first examine the performance of models that use heuristic rules such as inspecting the rate of change of glucose concentration levels. Dassau et al. [16] evaluated their meal detection algorithm on 17 subjects who consumed breakfast (22–105 g). The CGM sampling time interval is 1 min. The detection time from meal onset the ensemble method is reported to be 30 min. This discrepancy in sampling time and reported metrics makes a fair comparison difficult.

Samadi et al. [18] studied an in silico population of 30 individuals comprising 10 adults, 10 adolescents, and 10 children. The overall performance is reported to be 91.7% precision and 91.3% recall. The meal detection time is not provided but the meal estimate error is 23.1%. However, the performance of the adult cohort is more comparable with the study undertaken in this chapter. Due to the higher variability in adults the results are lower, with 79% precision, 87% recall, and 22% meal size error. This is further supported by a study with 11 adult clinical subjects that showed 93.5% recall and 79% precision with a detection time delay of 34 min on average [19]. Zheng et al. [23] report on average a 88% recall and 93.3% precision with a detection delay time of 26 min when evaluated on 100 in silico subjects. A meal size estimation error of 1.2 ± 3.6 g is reported, although this comparison is unfair as the meal sizes and size range evaluated on is relatively small (14–40.8 g).

Finally, Ramkissoon et al. [17] also evaluate their approach on 10 in silico adults. Their trade-off setting, which is meant balance between false positives (FP) and recall, demonstrates 82% recall and 38 min detection time from meal start time. The authors report a false positive rate of 0.2 per day, and from the reported data a mean 92.5% precision is determined. However this approach does not estimate meals for enabling postprandial control.

The proposed approach is more comparable to those of Xie and Wang [21] and Mahmoudi et al. [22] as these rely on uncertainty quantification and outlier detection to detect unannounced meals. Mahmoudi et al. [22] study 10 adult subjects from an in-silico cohort. The reported recall (99.5%) for this method is relatively higher than our proposed method. However, this comes at the expense of the detection time as this is longer at 58 min, and the precision is not reported. Xie and Wang [21] evaluate the VSD performance with 30 in-silico participants. The reported recall (76%) and precision (84%) are relatively lower than the our proposed method. In addition, the meal detection delay is further (45 min) than our proposed method and has worse meal estimate error. Both approaches use a Kalman filter to quantify uncertainty although this assumes the distribution of errors is Gaussian and may be the reason for the differences in performance. The primary difference in using multiple quantile regression as opposed to Kalman filters is that the assumption of normality is relaxed.

Given the importance of the precision and recall, we compare the algorithms using the F-score which is the harmonic mean of the two metrics. Our proposed method achieves an average F-score of 0.84 for both meals and snacks. Given that meals are the primary challenge for automated postprandial glucose control, we also consider that when our approach is solely evaluated on meals—as is done with other methods—it achieves the highest average F-score of 0.92.

### 5.2. Misestimation of Carbohydrate Content

One of the primary challenges in developing a suitable framework for detection and estimation of unannounced meals is the misestimation of carbohydrate content, either by underestimation or overestimation. As noted in Figure 2, the error in the model prediction varies, i.e., the median glucose prediction level is sometimes higher or lower than the true CGM signal. Consequently, the use of the mean absolute error of the validation results in the possibility of underestimation or overestimation of carbohydrate content during meal estimation. In addition, different meals and snacks tend to lead to different glucose rates of appearance when ingested which may lead to misestimation if the model only learns a single glucose rate of appearance. The effect of this limitation is partly addressed by the 90g limit on estimated meals at any instant which explains the concentrated horizontal line observed in Figure 4.

In future work, the extent of misestimation can be minimised by improving the predictive performance of the model. This may be achieved by incorporating physiological factors—for example, explicitly allowing different rates of appearance of glucose from different meals/snacks—in our current approach. This would have the subsequent effect of reducing the extent of misestimation of carbohydrate sizes by reducing the error in predicted glucose levels.

### 5.3. Safety Monitoring with Uncertainty Quantification

Safety is an important factor in the development of automated insulin delivery systems. Consequently, safety considerations are generally considered not only in the evaluation but in the development and deployment of such systems as well. A prominent challenge once such data driven models are deployed is that a scenario such as dataset shift can lead to sub-optimal performance [36]. Dataset shift occurs when there is a change in the conditions present in the training setting. This can be the case when the individual’s physiological state (e.g., menstrual cycle, illness, stress), behaviour, and/or the attached CGM sensor noise is different during deployment. The result of this can be increase in the number of false negatives or in the worse case of increased variability as noted in [19], an increase in false positives. This could increase potential risk of hypoglycaemia and lead to compromised control performance.

Although overcoming this remains an area of active research, our proposed method could provide an avenue to monitor the possible cases of significant distribution shift. In deployment, the prediction intervals could be monitored over successive periods (e.g., overnight) to detect if a significant deviation has occurred in their coverage. This is possible given that the computed aleatoric uncertainty is generally unaffected by significant distribution shifts [37]. This can serve as a marker for when the deployed model needs to be retrained in the event that the model is not robust to such distribution shifts. An alternative approach would be based on monitoring the epistemic uncertainty. Epistemic uncertainty captures the uncertainty stemming from input samples being different form samples the model was trained on in the training set [37]. In the case of epistemic uncertainty, no action would be undertaken in regions with high epistemic uncertainty. Ultimately, uncertainty quantification should be an essential component for this application area moving forward and form the basis of future work for building safe and reliable systems.

## 6. Conclusions

Current artificial pancreas systems are hybrid closed-loop controllers and therefore require the user to perform manual meal announcements in order to adequately handle postprandial hyperglycaemia. Although preprandial bolusing is shown to be most beneficial in achieving glycaemic targets, this places a cognitive burden on the user. Furthermore, the quality of glycaemic control is dependent on how well the individual is engaged with timely meal announcement and accurate carbohydrate estimation, which studies show is not always the case.

We develop a novel algorithm for meal detection and estimation of unannounced meals based on neural networks and multitask quantile regression. Compared to existing algorithms, this proposed approach achieves a better F-score for meal detection and competitive meal estimation performance based on simulation results. In addition, this algorithm provides a significant improvement in an artificial pancreas system to provide more effective closed-loop control. The hybrid closed-loop configuration shows better glycaemic control than our proposed approach, however, is worse at dealing with hypoglycaemia during control from the CVGA assessment. This study suggests that our proposed algorithm can serve as a viable approach for achieving fully automated closed-loop insulin delivery.

## Figures and Tables

**Table 1:** Cohort performance metrics for the meal detection performance for the different mealtimes. The meals section involves accounting for algorithm performance without snacks included.

Metric	CHO (g)				
	Breakfast	Lunch	Snack	Dinner	Overall (Meals)
	70 ± 7	100 ± 10	30 ± 3	80 ± 7	70 ± 27 (83 ± 15)
**Meal Detection Performance**					
Precision (%)	86 ± 7	98 ± 2	97 ± 5	94 ± 6	93 ± 4 (92 ± 4)
Recall (%)	90 ± 5	97 ± 3	24 ± 14	89 ± 4	76 ± 5 (92 ± 3)
Delay (min)	38 ± 13	36 ± 11	41 ± 23	37 ± 15	38 ± 15 (37 ± 13)

**Table 2:** A comparison of the performance in terms of glycaemic metrics and risk indices between different configurations of the BiAP: meal announcement (BiAP-MA), meal detection (BiAP-MD), and without either detection or announcement (BiAP-NMA). *p*-values calculated with Wilcoxon signed-rank test are underlined.

Metric	Controller			*p* *	*p* ^†^
	BiAP-MA	BiAP-MD	BiAP-NMA		
**Glycaemic Targets**					
Mean BG (mg/dL)	137.7 ± 5.0	144.5 ± 6.8	148.9 ± 9.8	0.002	0.003
TIR (%)	84.7 ± 5.1	77.8 ± 6.3	73.9 ± 7.9	0.002	0.0007
TAR (%)	13.7 ± 4.4	20.7 ± 6.0	24.9 ± 7.8	0.002	0.0009
TBR (%)	1.5 ± 1.3	1.4 ± 0.9	1.3 ± 1.2	0.8	0.4
**Risk Indices**					
HBGI	3.2 ± 0.8	4.3 ± 1.1	5.1 ± 1.5	0.002	0.0005
LBGI	0.5 ± 0.4	0.6 ± 0.4	0.5 ± 0.3	0.3	0.1
RI	3.7 ± 1.0	4.9 ± 1.3	5.6 ± 1.6	0.002	0.002

*p* * = BiAP-MD vs. BiAP-MA; *p* ^†^ = BiAP-MD vs. BiAP-NMA.

**Table 3:** A comparison of reported performance metrics in the literature for automatic meal detection and estimation algorithms with our proposed approach.

Algorithm	Performance Metrics					
	Precision	Recall	F-Score	Delay	Size Error	UQ
Dassau et al. [16]	-	-	-	30 min	-	**✗**
Ramkissoon et al. [17]	92.5%	82%	0.87	38 min	-	**✗**
Samadi et al. [18]	79%	87%	0.86	-	23%	**✗**
Samadi et al. [19]	79%	93.5%	0.86	35 min	-	**✗**
Zheng et al. [23]	93%	88%	0.91	26 min	-	**✗**
Xie and Wang [21]	84%	76%	0.80	45 min	43%	**✓**
Mahmoudi et al. [22]	-	99.5%	-	58 min	-	**✓**
Ours	93%	76%	0.84	38 min	31%	**✓**
Ours—Meals	92%	92%	0.92	37 min	19%	**✓**

UQ = Uncertainty Quantification.

## Abbreviations

The following abbreviations are used in this manuscript:

AP	Artificial Pancreas
BiAP	Bio-inspired Artificial Pancreas
BG	Blood Glucose
CGM	Continuous Glucose Monitor
CHO	Carbohydrate
CSII	Continuous Subcutaneous Insulin Infusion
CV	Coefficient of Variation
CVGA	Control-variability grid analysis
DNN	Deep Neural Network
EKF	Extended Kalman Filter
FN	False Negative
FP	False Positive
HBGI	High Blood Glucose Index
HbA1C	Glycated Haemoglobin
LBGI	Low Blood Glucose Index
LSTM	Long Short-Term Memory
MAE	Mean Absolute Error
PI	Prediction Interval
RI	Risk Index
RMSE	Root Mean Square Error
Seq2Seq	Sequence-to-Sequence
T1D	Type 1 Diabetes
TN	True Negative
TP	True Positive
TIR	% Time In Range
TAR	% Time Above Range
TBR	% Time Below Range
UKF	Unscented Kalman Filter
VSD	Variable State Dimension

## Appendix A

The model architecture is primarily a multitask encoder-decoder architecture based on LSTM recurrent neural networks. These models are trained on 80% of the training dataset and validated on the remaining 20%. The predictive performance of the model is evaluated using the RMSE, MAE, and PI coverage (pre-specified at 95%). Table A1 shows the performance for each individual.

**Table A1:** Performance metrics for neural network on validation set: The parameters of the meal detection and estimation framework are selected based on performance of the model on the validation set. This is based on last 20% of the training data in order to attain the predictive accuracy and prediction interval necessary.

ID	Metric		
	RMSE	MAE	PI
1	5.8	4.0	95.3
2	5.9	4.1	94.5
3	8.0	4.6	94.7
4	5.7	4.0	94.9
5	7.7	4.3	93.6
6	5.7	4.1	94.9
7	6.7	4.8	94.7
8	7.2	4.3	93.7
9	5.0	3.7	97.3
10	8.4	4.3	95.4
Average	6.6 ± 1.1	4.2 ± 0.3	94.9 ± 1.0
